# Novel dithiocarbamate derivatives are effective copper-dependent antimicrobials against *Streptococcal* species

**DOI:** 10.3389/fmicb.2022.1099330

**Published:** 2023-01-20

**Authors:** Sanjay V. Menghani, Yamil Sanchez-Rosario, Chansorena Pok, Renshuai Liu, Feng Gao, Henrik O’Brien, Miranda J. Neubert, Klariza Ochoa, Meredythe Durckel, Riley D. Hellinger, Nadia Hackett, Wei Wang, Michael D. L. Johnson

**Affiliations:** ^1^Department of Immunobiology, University of Arizona College of Medicine - Tucson, Tucson, AZ, United States; ^2^Medical Scientist Training MD-PhD Program (MSTP), University of Arizona College of Medicine - Tucson, Tucson, AZ, United States; ^3^Department of Microbial Pathogens and Immunity, Rush University, Chicago, IL, United States; ^4^Department of Pharmacology and Toxicology, R. Ken Coit College of Pharmacy, University of Arizona, Tucson, AZ, United States; ^5^Valley Fever Center for Excellence, University of Arizona College of Medicine - Tucson, Tucson, AZ, United States; ^6^BIO5 Institute, University of Arizona College of Medicine - Tucson, Tucson, AZ, United States; ^7^Asthma and Airway Disease Research Center, University of Arizona College of Medicine - Tucson, Tucson, AZ, United States

**Keywords:** copper, antimicrobial, dithiocarbamates, ICP-OES, animal model, *Streptococcus*

## Abstract

Despite the availability of several vaccines against multiple disease-causing strains of *Streptococcus pneumoniae*, the rise of antimicrobial resistance and pneumococcal disease caused by strains not covered by the vaccine creates a need for developing novel antimicrobial strategies. *N,N-dimethyldithiocarbamate* (DMDC) was found to be a potent copper-dependent antimicrobial against several pathogens, including *S. pneumoniae*. Here, DMDCs efficacy against *Streptococcal* pathogens *Streptococcus pyogenes*, *Streptococcus agalactiae*, and *Streptococcus anginosus* was tested using bactericidal and inductively coupled plasma - optical emission spectrometry. After confirming DMDC as broad-spectrum streptococcal antimicrobial, DMDC was derivatized into five compounds. The derivatives’ effectiveness as copper chelators using DsRed2 and as copper-dependent antimicrobials against *S. pneumoniae* TIGR4 and tested in bactericidal and animal models. Two compounds, sodium *N*-benzyl-*N*-methyldithiocarbamate and sodium *N*-allyl-*N*-methyldithiocarbamate (herein “Compound 3” and “Compound 4”), were effective against TIGR4 and further, D39 and ATCC® 6303™ _(a type 3 capsular strain). Both Compound 3 and 4 increased the pneumococcal internal concentrations of copper to the same previously reported levels as with DMDC and copper treatment. However, in an *in vivo* murine pneumonia model, Compound 3, but not Compound 4, was effective in significantly decreasing the bacterial burden in the blood and lungs of *S. pneumoniae*-infected mice. These derivatives also had detrimental effects on the other streptococcal species. Collectively, derivatizing DMDC holds promise as potent bactericidal antibiotics against relevant streptococcal pathogens.

## Introduction

*Streptococcus pneumoniae* (also known as the pneumococcus) is a Gram-positive bacterium that is a leading cause of pneumonia, otitis media, meningitis, and sepsis. As such, the pneumococcus causes a significant disease burden in the pediatric and elderly populations despite effective vaccines against the most common strains (Zintgraff et al., 2021). Normally a commensal of the nasopharynx and upper respiratory tract, the causes and gene regulation of pathogenic transformation is an ongoing topic of investigation (Sorg et al., 2020). In addition, the United States Centers for Disease Control and Prevention (CDC) has identified drug-resistant *S. pneumoniae* as a serious threat in their 2019 report “Antibiotic Resistance Threats in the United States,” with approximately 30% of clinical strains of *S. pneumoniae* displaying resistance to at least one antibiotic and many more harboring multidrug resistance (CDC, 2019).

The global rise of antibiotic-resistant bacterial strains has created a renewed desire to develop novel antimicrobials. Traditionally, most antibiotics used clinically against *Streptococcus pneumoniae* have targeted a single bacterial protein or enzyme for bactericidal or bacteriostatic effect, like the bacterial cell wall for β-lactams or bacterial RNA translation for aminoglycosides (Domalaon et al., 2018; Sibinelli-Sousa et al., 2021). Unsurprisingly, the global selective pressure of using single-target antibiotics allows bacteria to escape antibiotic coverage by acquiring fewer mutations. Clinically relevant resistant strains are seen within years of the introduction of novel antibiotics, as was observed for penicillin and methicillin resistance in the bacteria *Staphylococcus aureus* in the mid-1940s (Harkins et al., 2017; Turner et al., 2019). Beyond the less than 25 bacterial proteins and enzymes inhibited by these traditional antibiotics, computational studies have identified 300 essential and highly conserved antimicrobial targets to advance drug discovery (Ji et al., 2001; Forsyth et al., 2002). A drug targeting multiple bacterial enzymes at once would require a bacterium to acquire multiple mutations to develop antibiotic resistance (Fair and Tor, 2014). Another strategy for developing novel antimicrobials is drug repurposing, testing the *in vitro* antimicrobial activity of molecules or drugs that have United States Food and Drug Administration (FDA) approval for other indications (Farha and Brown, 2019). The recent identification of antimicrobial properties of the drug disulfiram has combined these two principles (Dalecki et al., 2015; Hu et al., 2020; Potula et al., 2020; Shirley et al., 2021).

The drug disulfiram (also known as tetraethylthiuram disulfide, TETD, or the brand name Antabuse) has been used to treat alcohol dependence for decades, despite a significant decrease in clinical use over time. In the body, disulfiram is rapidly converted to its metabolite diethyldithiocarbamate (DETDC), which is complexed with divalent metal zinc and copper ions (Johansson, 1992). Recently, there has been a growing body of evidence for the antimicrobial properties of disulfiram’s metabolites. In 2020, a group led by Ghosh et al. identified Zn^2+^-diethyldithiocarbamate as a potent antiparasitic agent against *Entamoeba histolytica* and other parasites (Ghosh et al., 2020). In 2021, our group performed a targeted antimicrobial drug screen involving dithiocarbamate compounds (Menghani et al., 2021). In that study, diethyldithiocarbamate (DEDC) and *N,N*-dimethyldithiocarbamate (DMDC) were investigated, with DMDC being identified as a potent copper-dependent antimicrobial against *S. pneumoniae*, *Coccidioides immitis*, and *Schistosoma mansoni* (Menghani et al., 2021). Since this study, other groups have also identified copper-dependent antibiotic potential for dithiocarbamates (Chen et al., 2022).

Mechanistically, DMDC significantly increases the intrabacterial concentration of copper, putting stress on the bacteria to export copper before mismetallation inactivates or reduces the efficiency of key bacterial enzymes, eventually leading to bacterial death (Johnson et al., 2015; Menghani et al., 2022). Further, when the excess oxidized copper enters the bacteria, it diminishes the reductive capacity of the bacteria, which must reduce copper ions in order to export them (Rensing et al., 2000; Fan and Rosen, 2002; Singleton et al., 2008; Zhou et al., 2012a,b; Menghani et al., 2022). As a result, the pneumococcus cannibalized its capsule, making macrophage mediating killing more efficient, and presumably to extract electrons from the sugar building blocks to maintain the intrabacterial reducing environment (Benedict, 1911; Menghani et al., 2022).

The *Streptococcus* genus has many significant pathogens. Most notably are *Streptococcus pyogenes* (group A strep, cause of strep throat, impetigo and necrotizing fasciitis)*, Streptococcus agalactiae* (group B strep, cause of fatal neonatal infections) and *Streptococcus anginosus* (cause of pyogenic infections and an emerging pathogen in vaginal dysbiosis) (Tao et al., 2019; Jiang et al., 2020). While due to increasing isolations and competence, but still being rare, antibiotic resistance is not a current problem with *S. anginosus* (Bauer et al., 2018; Leszczynski et al., 2019), however, it is a significant issue with *S. pyogenes* and *S. agalactiae* (Rubio-Lopez et al., 2012; Haenni et al., 2018; Barkowsky et al., 2019; Li et al., 2020; Kawaguchiya et al., 2022).

In this study, DMDC was successfully able to kill different Streptococcal species and because of this, dithiocarbamate compounds were then derived from the starting compound, DMDC. Derivatives tested the *in vitro* copper-dependent antimicrobial efficacy of these compounds (named “Compound 1” through “Compound 5”) against *S. pneumoniae* TIGR4 (a virulent strain), D39 (a virulent derivative of the Avery strain), and Type 3 (a hyper-encapsulated strain). The zinc-dependent toxicity of these compounds was also tested against TIGR4 and found to be bacteriostatic. Next, the *in vivo* efficacy of these compounds was tested in a murine pneumonia model demonstrating that they reduced the bacterial burden in the lungs. Finally, the ability of these compounds to enhance *in vitro* macrophage-mediated killing was tested, finding the combination of Compound 3 and copper to be cleared more efficiently. Overall, this study identifies novel compounds in the dithiocarbamate class that are broadly anti-streptococcal *in vitro* and effective *in vivo*.

## Materials and methods

### Synthesis of DMDC analogs

The general synthetic procedure used was with a solution of KOH (Sigma-Aldrich, United States; 56 mg, 1.00 mmol) in EtOH (Sigma-Aldrich, United States; 10 ml) was cooled to 0°C, then morpholine (Sigma-Aldrich, United States; 87 μl, 1.00 mmol) and CS_2_ (Sigma-Aldrich, United States; 151 μl, 2.50 mmol) were added to the solution successively. The resulting mixture was stirred at room temperature for 2 h, and then the solvent was reduced under vacuum. The first compound, Compound 1 (Dipotassium piperazine-1,4-dicarbodithiate, LRS01-057), was synthesized. ^1^H NMR (400 MHz, DMSO-*d*_6_) δ 4.34–4.22 (m, 4H), 3.51–3.39 (m, 4H). ^13^C NMR (100 MHz, DMSO-*d*_6_) δ 215.02, 66.58, 50.01.

Following the general procedure, the title compound was synthesized in 57% as a white solid to Compound 2 (Sodium 4-(*p*-tolyl)piperazine-1-carbodithioate, LRS01-084) ^1^H NMR (400 MHz, DMSO-*d*_6_) δ 4.20 (s, 8H). ^13^C NMR (100 MHz, DMSO-*d*_6_) δ 214.14, 49.51.

Following the general procedure using NaOH (Sigma-Aldrich, United States) as a base, the title compound was synthesized in 83% as a light yellow solid to synthesize Compound 3 (Sodium *N*-benzyl-*N*-methyldithiocarbamate, LRS01-075) ^1^H NMR (400 MHz, DMSO-*d*_6_) δ 6.98 (d, *J* = 8.1 Hz, 2H), 6.80 (d, *J* = 8.2 Hz, 2H), 4.41 (t, *J* = 5.1 Hz, 4H), 2.96 (t, *J* = 5.1 Hz, 4H), 2.16 (s, 3H). ^13^C NMR (100 MHz, DMSO-*d*_6_) δ 214.73, 149.41, 129.78, 127.97, 116.15, 49.25, 49.09, 20.49.

Following the general procedure using NaOH as a base, the title compound was synthesized in 73% as a white solid for Compound 4 (Sodium *N*-allyl-*N*-methyldithiocarbamate, LRS01-077). ^1^H NMR (400 MHz, DMSO-*d*_6_) δ 7.28–7.19 (m, 4H), 7.19–7.12 (m, 1H), 5.43 (s, 2H), 3.24 (s, 3H). ^13^C NMR (100 MHz, DMSO-*d*_6_) δ 215.70, 139.58, 128.42, 127.66, 126.75, 57.85, 40.99.

Following the general procedure using NaOH as a base, the title compound was synthesized in 56% as a white solid to Compound 5 (Sodium ((*2S*,*3S*)-1-ethoxy-3-methyl-1-oxopentan-2-yl) carbamodithioate, LRS01-072). ^1^H NMR (400 MHz, DMSO-*d*_6_) δ 5.83–5.70 (m, 1H), 5.08–5.02 (m, 1H), 5.02–4.97 (m, 1H), 4.73 (d, *J* = 5.8 Hz, 2H), 3.25 (s, 3H). ^13^C NMR (101 MHz, DMSO-*d*_6_) δ 214.76, 135.06, 116.16, 57.57, 40.88.

### Bacterial culture

Strains used here are *S. pneumoniae* TIGR4, D39, and Type 3, *S. pyogenes* M1, *S. agalactiae* ATCC 13813, and *S. anginosus* (Andrewes and Horder) Smith and Sherman emend. Whiley and Beighton 33,397™. For *S. pneumoniae* TIGR4, D39, and Type 3, *S. agalactiae* ATCC 13813, and *S. anginosus* M17 media (M17; BD Difco, United States) was prepared according to the manufacturer’s instructions. Briefly, 37.25 g of powder was suspended in 950 ml of Milli-Q grade water (≥18.0 MΩ cm^−1^) and autoclaved at 121°C for 15 min before cooling to 50°C and adding 50 ml of a sterile 10% lactose (Beantown Chemical, United States) solution. For *S. pyogenes*, the culture medium was grown in Difco Todd Hewitt Broth (ThyB). For plating, Tryptic Soy Agar (TSA; Hardy Diagnostics, United States) was dissolved in Milli-Q water and autoclaved. After cooling autoclaved TSA, 5% defibrillated sheep’s blood (HemoStat Laboratories) of final volume and 20 μg/ml neomycin (Research Products international, United States) was added to the solution (for *S. pneumoniae* TIGR4 only). These plates (blood agar plates—BAP) were used for routine culture on solid media and “killing curve” serial dilution CFU counting. Bacteria from freshly streaked plates were placed into M17 and grown at 37°C in 5% CO_2_, to an optical density (OD or OD_600_) of 0.125 for growth curve assays and to an OD of ~0.300 for killing curve assays. To prepare working stocks, growing cultures are resuspended in fresh media +20% v/v glycerol and stored at –80°C. Aliquot viability and CFU counts were determined before use in experiments. Glycerol stock aliquots were diluted 1:5 into M17 with indicated copper and compound concentrations for assays.

### Growth curves

Growth curve assay as described in Menghani et al. (2021). Briefly, copper stock solutions at 100 mM were prepared from CuSO_4_ pentahydrate (VWR Life Sciences, United States) in Milli-Q water. Stock solutions of 100 mM Zn^2+^ were prepared from ZnSO_4_ heptahydrate (VWR Life Sciences, United States). Stock solutions of 1 mM Compound compounds were prepared in Milli-Q water from solute derived and purified by the University of Arizona Center for Drug Discovery (ACDD). Sterile, individually wrapped clear 96 well polystyrene plates (Greiner Bio-One, USA) were arranged to test a range of concentrations combinations of Cu^2+^, Zn^2+^, and compounds. Frozen aliquots of bacteria were thawed and diluted five-fold into fresh M17 before adding 20 μl per well into a total well volume of 200 μl (1:50 total dilution). Assay plates were loaded into a Biotek Cytation5 (Biotek, Vermont, United States) pre-equilibrated to 37°C and 4% CO_2_. Gas control settings were modified for an elevation of 720 m according to the manufacturer’s directions. The protocol-maintained temperature and CO_2_, while measuring OD absorbance at 600 nm every 30 min for 16–20 h.

### Killing curves

Killing curve assay as described in Menghani et al. (2021). Aliquots of bacteria were thawed and diluted 10-fold into assay conditions prepared in M17 media. Assay conditions included various concentrations of CuSO_4_, Compound compounds, Zn^2+^, and Mn^2+^ (Sigma-Aldrich, United States). After exposure to the indicated conditions, bacteria were incubated at 37°C in 5% CO_2_ for the stated time. Samples were serially diluted, plated on BAP, incubated overnight at 37°C in 5% CO_2_, and counted to determine viable CFU. Colonies on each plate were counted and multiplied by the appropriate dilution factor based on which dilution it was to determine CFU/ml. For plates in which no colonies were visualized, this was deemed to be below the limit of detection (LoD) and is noted with a data point below the LoD line.

### Protein purification

As performed previously (O’Brien et al., 2020) BL21 cells were transformed with DsRed2-pBAD and grown overnight in a shaker incubator at 250 rpm at 37°C on LB with ampicillin (Sigma-Aldrich, United States) 150 μg/ml. In the morning, the culture was centrifuged and resuspended on fresh media. Terrific broth (100 μg/ml amp) was inoculated to an optical density of 0.02. The culture was incubated at 37°C in a shaker incubator, and growth was monitored until 0.5–0.6 optical density, at which point the culture was induced with 0.1% arabinose (Tokoyo Chemical Industry), and the temperature was reduced to 16°C overnight. After 16–18 h, the culture was centrifuged, and the pellet was collected. The pellet was resuspended in buffer 50 mM TRIS (Sigma-Aldrich, United States) pH 7.4, 500 mM NaCl, 25 mM imidazole (Sigma-Aldrich, United States), 5% glycerol (RPI, United States), and lysed by sonication after adding protease cocktail of Leupeptin (Sigma-Aldrich, United States), Pepstatin A (Sigma-Aldrich, United States), phenylmethylsulfonyl fluoride or PMSF (Sigma-Aldrich, United States) and 0.2 mg/ml DNase (Sigma-Aldrich, United States). The lysate was centrifuged to remove debris, and the cell lysate was purified on an AKTA Pure *via* an affinity nickel column and buffer exchanged *via* a desalting column. The fractions were concentrated using a 10,000 mw CO (Millipore). The protein was left to mature overnight at room temperature. Concentration was calculated by sequenced and absorbance at 280 nm. Purity was assessed by 12% SDS PAGE. The protein was stored at 4°C and used within 2 weeks.

### Competition assay

A relative affinity test was performed using the reversible fluorescence quenching of DsRed2 by copper (Eli and Chakrabartty, 2006; Sumner et al., 2006). The following was added to a 96 well plate (Greiner bio-one 655,096) in a total volume of 200 μl: 400 μM of freshly dissolved compound was added in 10 mM MOPS (Sigma-Aldrich, USA) pH 7.2 (chelated overnight with a Chelex 100 Biorad, United States and filtered sterilized through 0.2 μm membrane), 20 μM DsRed2, 100 μM copper (CuSO_4_). In a Biotek Cytation5, the absorbance at 585 nm was monitored for 3 h to establish chelation of copper by the compounds and restore DsRed2 fluorescence.

### Inductively coupled plasma optical emission spectroscopy

To measure the internal metal concentration of the bacteria, strains were initially cultured on M17 + 5 mM lactose and frozen at −80°C in 20% glycerol. These glycerol stocks were used as the seed stock to inoculate 150 ml of M17 + 5 mM lactose. The bacterial culture was incubated at 37°C under 5% CO_2_ until an OD of ~0.400 was reached. The culture was split into the indicated treatments and control. Incubation with treatments was performed at 37°C and 5% CO_2_ for 30 min. Samples were quenched in a −3°C water bath to slow metabolism, followed by two washes of cold buffer (Tris 50 mM, NaCl 150 mM, EDTA 50 mM at pH 7.6), and centrifugation 3,500 ×*g* for 10 min at 4°C. Cold decanted samples were resuspended in 70% HNO_3,_ followed by overnight incubation at 65°C. After incubation, the samples were diluted to 2.5% HNO_3_ using Milli-Q water at 18.1 MΩ. Bacterial plate counts were performed in TSA + 5% Sheep’s Blood through serial dilutions, as described above. Samples were analyzed for metal content using an iCAP PRO XDUO ICP-OES with a wavelength 324.8 nm copper. Standards were made using the iCap Series Multi-element test solution ICAP 6000 series Validator from Thermo scientific, and metal content of the washed samples was calculated using the Qtegra software. Experiments were performed in triplicate.

### Animal experiments

All mouse studies were conducted with prior approval and under the guidelines of the IACUC at the University of Arizona (IACUC protocol number 18-410, R35 GM128653) as previously performed (Menghani et al., 2021, 2022). All mice were maintained in a biosafety level 2 (BSL2) animal facility and monitored daily for signs of moribund. Eight-week-old female BALB/cJ mice (Jackson Laboratory) were anesthetized with 3% isoflurane and intranasally infected with an inoculum of 10^7^ CFU viable *S. pneumoniae* in 25 μl of Tris-buffered saline (TBS; 50 mM Tris, 150 mM NaCl, pH 7.4). Cohort controls were given 25 μl of TBS. At 4-, 8-, or 24-h post-infection, mice were treated with doses of intranasal Compound 3 or Compound 4 (approximately 1.6 mg/kg) in 25 μl of TBS. Mice were sacrificed by CO_2_ asphyxiation and immediately dissected for lung and blood collection 48 h post-infection. Lung tissue was collected into 1.5 ml tubes containing 500 ml of phosphate-buffered saline (PBS; Gibco, United States) after a brief initial wash in 500 μl of PBS to remove any excess blood during dissection. The tissue was then homogenized and centrifuged for 30 s at 400 ×*g*. Blood samples (5 μl volume) were placed in a 45-μl volume PBS solution with heparin (10 UI/ml). Both lung and blood samples were then serially diluted at 1: 10 and plated on blood agar plates and incubated overnight at 37°C and 5% CO_2_ for growth. The resulting bacterial colonies were counted for quantification and comparison.

### Macrophage killing assays

J774A.1 macrophages (ATCC, United States) were maintained in a 37°C, 5% CO_2_ incubator with Dulbecco’s Modified Eagle’s Medium (DMEM; Sigma-Aldrich, United States) containing fetal bovine serum (FBS [10%vol/vol]; Sigma-Aldrich, United States), glutamine (2 mM; Sigma-Aldrich, United States), penicillin (50 units/ml; Sigma-Aldrich, United States), streptomycin (50 mg/ml; Sigma-Aldrich, United States), and NaHCO_3_ (0.015%). Cells were grown to 90% confluence in 12-well tissue culture plates (Greiner CELLSTAR®, Greiner Bio-One, United States) in 1 ml/well of growth medium. On the morning of the experiment, macrophages were washed twice with 1 ml PBS, and resuspended in 1 ml of “Serum-Free DMEM” growth medium without antibiotics, glutamine, NaHCO_3_ or FBS but supplemented with 5 ng/ml IFN-γ (Bio Basic, United States), 400 ng/ml LPS (EMD Millipore, MilliporeSigma, United States) for “priming of macrophages” experiment. For “*post hoc* killing efficiency” experiment, macrophages were resuspended in 1 ml of Serum-Free DMEM containing 32 μM Compound 3, 5 ng/ml IFN-γ, and 400 ng/ml LPS.

Macrophages were incubated at 37°C, 5% CO_2_ for 12 h. Glycerol stocks of TIGR4 *S. pneumoniae* kept at OD 0.3 are removed from-80°C storage, diluted into four 15 ml conical tubes of 5 ml total M17 + Lactose containing no additives (“Untreated”), 32 μM Compound 3, 250 μM CuSO_4_, and 250 μM CuSO_4_ + 32 μM Compound 3, respectively, for pre-treatment of bacteria experiment. Prior to incubation of bacteria in 37°C, 5% CO_2_, an inoculum plate is made by serial diluting 100 μl from the no additives conical. After 15 min of incubation, bacteria are centrifuged at 4500 ×*g* for 10 min and resuspended in DMEM without antibiotics, glutamine, NaHCO_3_ or FBS. Macrophages are removed from incubation, media is removed, washed with 1 ml PBS twice and then infected with 100 μl of *S. pneumonia*e solutions for both experiment types, corresponding to a multiplicity of infection (MOI) of 10 bacteria per macrophage. The 12-well tissue culture plates were centrifuged at 200 ×*g* for 2 min to facilitate co-culturing.

Wells were then washed twice with PBS at the given time-points (5, 15, and 30 min); each wash was followed by a 5-min incubation in a 37°C, 5% CO_2_ incubator in DMEM containing gentamicin (50 μg/ml). Macrophages were lysed in 0.02% SDS by pipetting up and down in ddH_2_O and serially diluted to determine the counts of viable intracellular bacteria. Data were normalized to the level of killing observed for the untreated TIGR4 bacteria for each assay.

### Statistical analysis

Statistical significance was analyzed using Student *t*-test (two-tailed, unpaired), two-way ANOVA, or one-way ANOVA and Dunnett’s multiple comparisons test (Prism 9.20; GraphPad Software, United States). The *p*-values were as follows: *p < 0.05, **p < 0.01, ***p < 0.001, and ****p < 0.0001.

## Results

### Streptococcal species are susceptible to copper dependent DMDC toxicity

**Figure 1:**
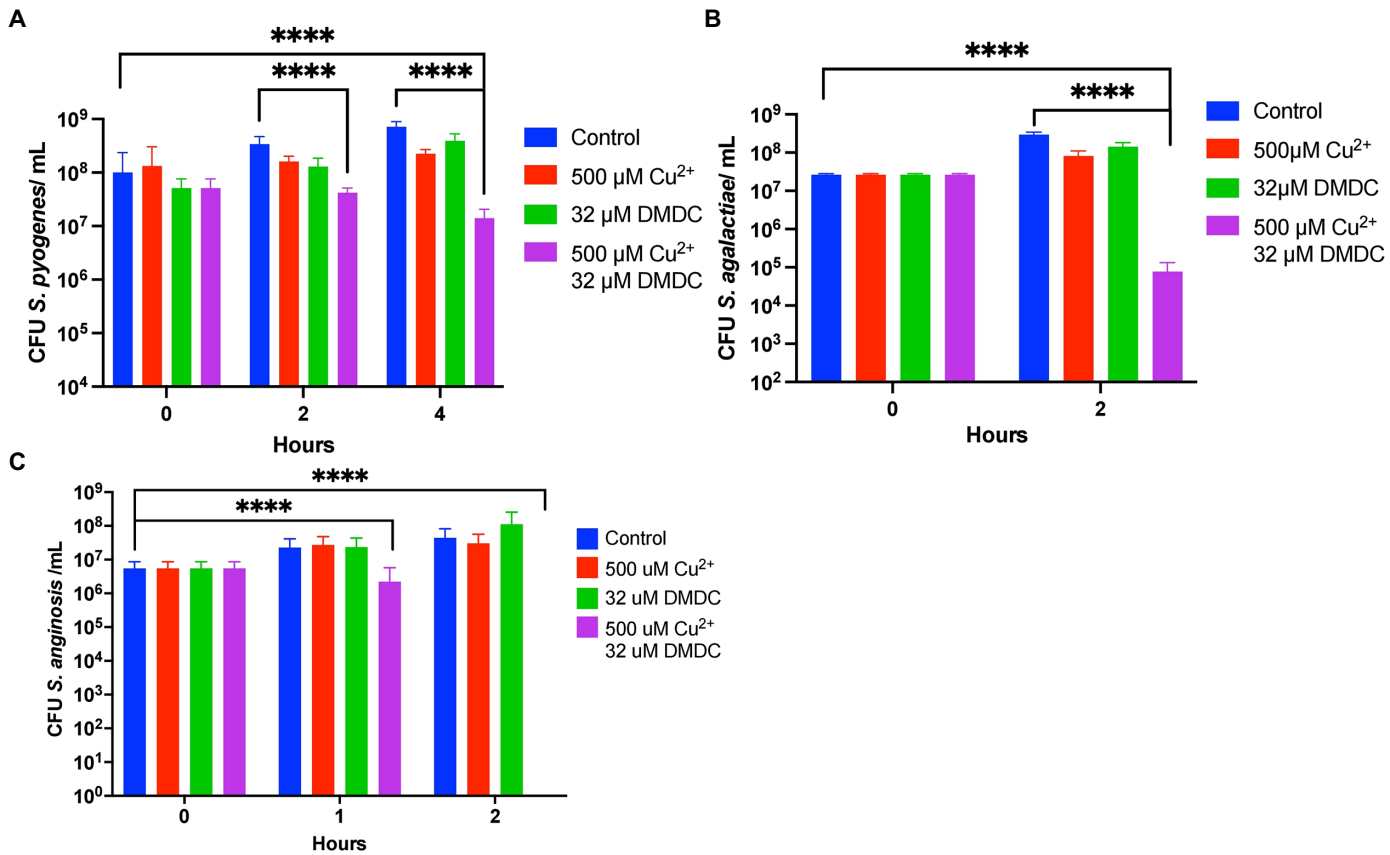
*In vitro* testing of copper-dependent antimicrobial activity of N,N-dimethyldithiocarbamate (DMDC) against different pathogenic Streptococcal species. Killing curve assays depicting the effects of DMDC and copper against *Streptococcus anginosus* **(A)**, *Streptococcus agalactiae* **(B)**, and *Streptococcus pyogenes* **(C)**. All bars indicate mean ± SD with *n* ≥ 3 across three independent replicates. Statistical differences were measured by one way ANOVA; ns, not significant, *p < 0.05, **p < 0.01, ***p < 0.001, and ****p < 0.0001.

**Figure 2:**
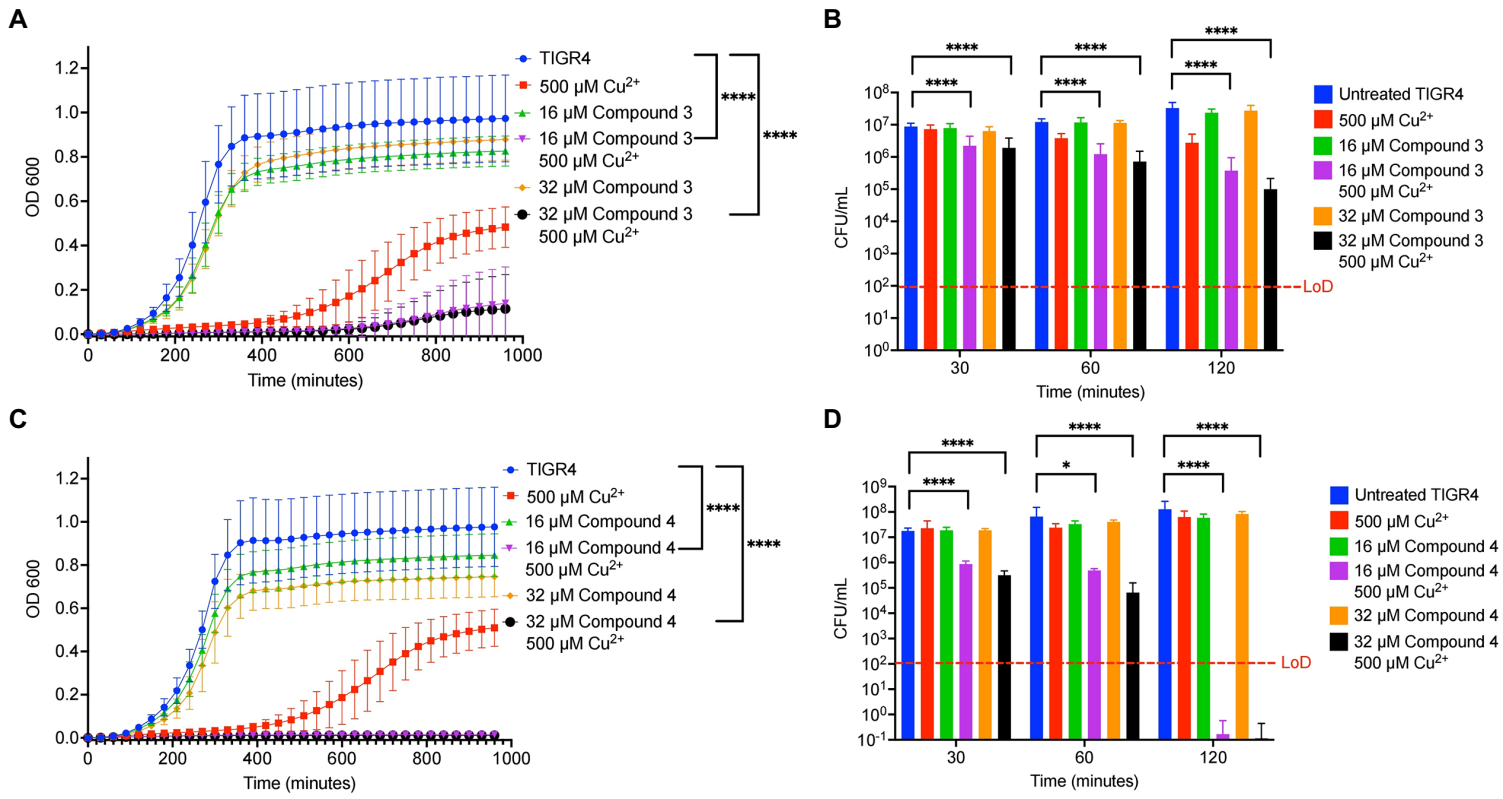
*In vitro* testing of copper-dependent antimicrobial activity of dithiocarbamate compounds derived from N,N-dimethyldithiocarbamate (DMDC). **(A)** Growth curve assay of *Streptococcus pneumoniae* TIGR4 exposed to the indicated concentrations of copper sulfate and Compound 3. **(B)** Killing curve assay of *S. pneumoniae* TIGR4 starting with an inoculum of 5.6×10^6^ CFU/mL in M17 media supplemented with indicated concentrations of copper sulfate and/or Compound 3. **(C)** Growth curve assay of *S. pneumoniae* TIGR4 exposed to the indicated concentrations of copper sulfate and Compound 4. **(D)** Killing curve assay of *S. pneumoniae* TIGR4 starting with an inoculum of 1.3 × 10^6^ CFU/ml in M17 media supplemented with indicated concentrations of copper sulfate and/or Compound 4. All bars indicate mean ± SD with *n* = 12–18 across 3 independent replicates for growth curves and *n* = 9 across 3 independent replicates for killing curves. Statistical differences were measured by one way ANOVA; ns, not significant, *p < 0.05, **p < 0.01, ***p < 0.001, and ****p < 0.0001.

DMDC has known copper dependent toxicity against *S. pneumoniae* TIGR4, D39 and Type 3 (Menghani et al., 2021, 2022). Before investigating possible DMDC derivatives against the pneumococcus alone, it was tested if DMDC and copper was an effective broader spectrum treatment against known pathogenic streptococcal strains *S. anginosus*, *S. pyogenes*, and *S. agalactiae* as a “go/no go” checkpoint. DMDC with copper had significant bactericidal effect (at least a 90% decrease) after 4 h with *S. pyogenes* (Figure 1A) and 2 h with *S. anginosus* and *S. agalactiae* (Figures 1B,C). The effect of DMDC with copper against *S. anginosus* and *S. agalactiae* were the closest in comparison to *S. pneumoniae,* for which DMDC killed roughly 3 logs of bacteria at the two-hour time point, however, *S. pyogenes* was slower to die taking 4 h constantly to get to 1 log of killing. Therefore, with the potential for treatment of streptococcal species, and previously reported fungi and schistosomes (Menghani et al., 2021), derivatizing DMDC proceeded.

### DMDC derivatives are effect at intoxicating *Streptococcus pneumoniae* TIGR4 with copper

DMDC was derived into five compounds that retain putative copper ionophore properties (Table 1). The five derived compounds were named “Compound 1” through “Compound 5.” Each compound was initially screened by performing *in vitro* growth curves to determine if a growth defect was associated with either the compound alone or in combination with copper. These bacteria were started at lag phase growth. Next, successful compounds were tested in killing curves to determine if there was a bactericidal or bacteriostatic effect of copper, compound, or combination treatment. These curves were done on bacteria at log phase growth. While it is more beneficial to eliminate the bacteria than to just restrict growth, the growth curves offer an entry point to analyze compounds that use less resources that the killing curves and correlate with the killing curves. Compound 1, Compound 2, and Compound 5 had no effect, and thus, experiments with these compounds were discontinued (data not shown). Compound 3 and Compound 4 showed statistically significant growth defects for the combination treatments in comparison to an untreated control in TIGR4 *S. pneumoniae* (Figures 2A, C). Further, both Compound 3 and Compound 4 showed significant bactericidal activity in killing curves with 2 or more logs for each (99% killing; Figures 2B, D). From these data, it was concluded that Compound 3 and Compound 4 are viable copper-dependent bactericidal antibiotics that warrant further investigation.

**Table 1:** Summary of screening results of each compound derived from DMDC Structure of each compound, growth curve assay results, and killing curve assay results are summarized for each compound.

**Compound**	**Structure**	**IUPAC name**	**Growth defect on growth curve**	**Killing curve effect**
Compound 1	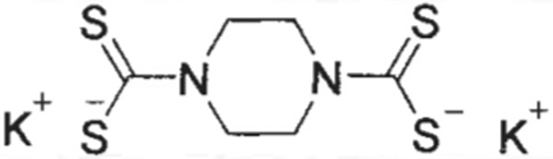	Dipotassium piperazine-1,4-dicarbodithiate	No	None
Compound 2	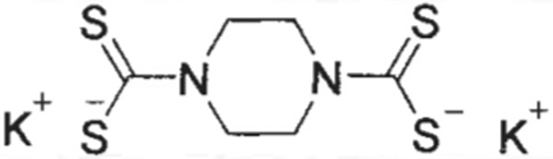	Sodium 4-(*p*-tolyl)piperazine-1-carbodithioate	No	None
Compound 3	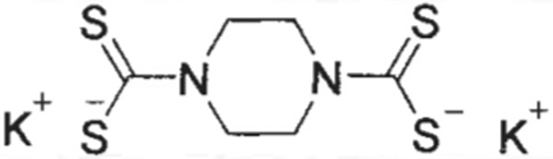	Sodium *N*-benzyl-*N-methyldithiocarbamate*	Yes	Bactericidal
Compound 4	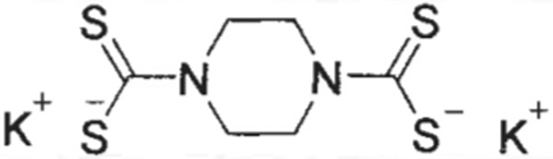	Sodium *N*-allyl-*N*-methyldithiocarbamate	Yes	Bactericidal
Compound 5	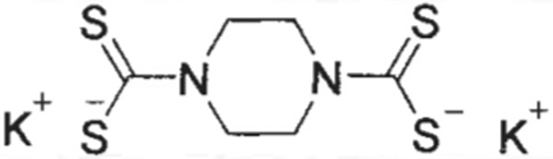	Sodium ((*2S*,*3S*)-1-ethoxy-3-methyl-1-oxopentan-2-yl)carbamodithioate	No	None

**Figure 3:**
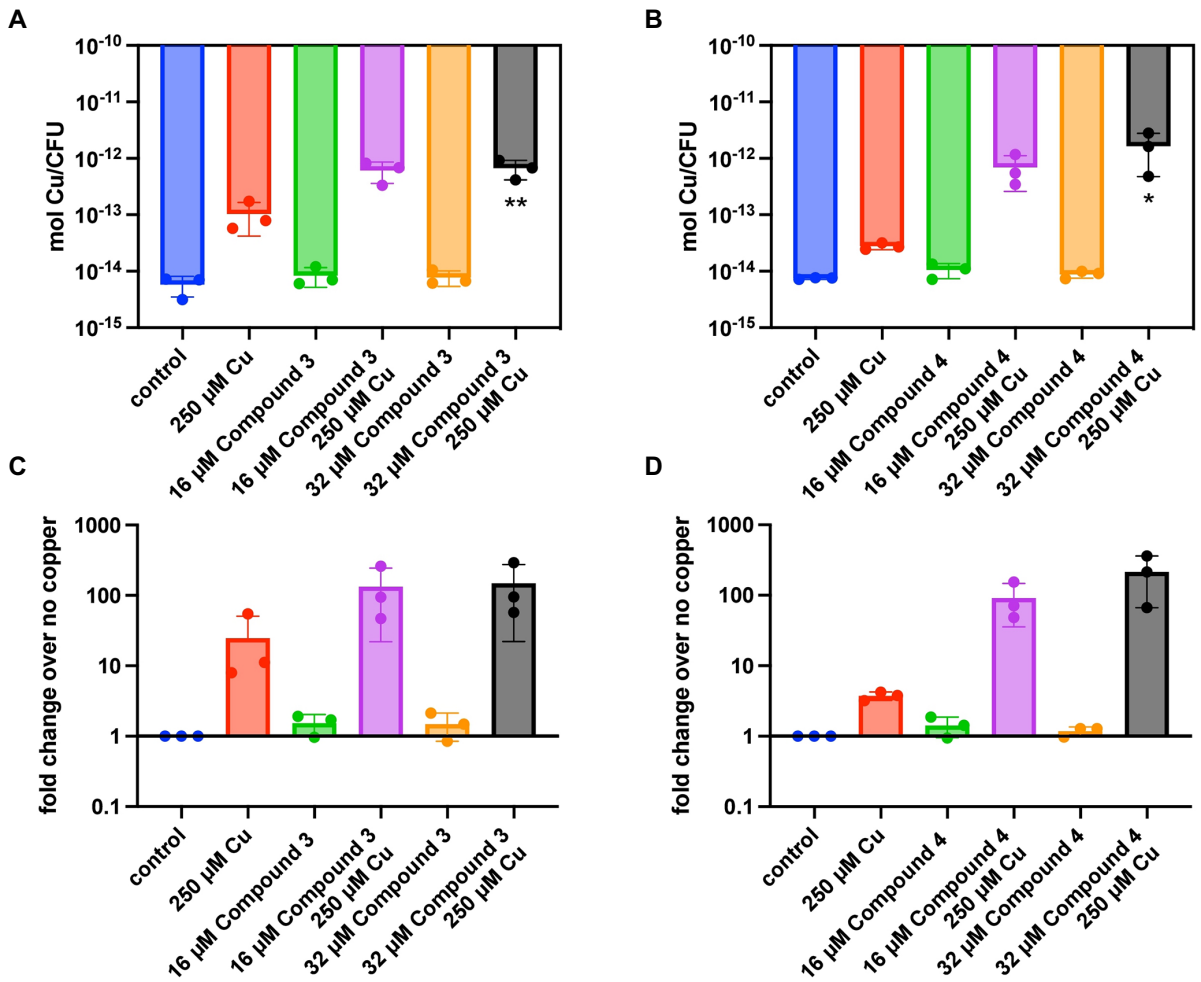
Intracellular copper concentration of TIGR4 after treatment with compounds and copper. Exponentially growing bacteria were exposed to a combination of compound and copper for 30 min. ICP-OES was used to measure intracellular copper. **(A,B)** represent moles of copper per CFU for Compounds 3 and 4 while **(C,D)** represent the fold change over the parent strain with nothing added. Experiments were performed in triplicate with statistical significance determined by an Ordinary one-way ANOVA; *p < 0.05, **p < 0.01.

The ability of the compounds to increase the concentration of copper inside TIGR4 was examined next. It was previously observed using DMDC plus copper that there was a significant increase in internal copper relative to copper or DMDC treatments alone (Menghani et al., 2022). Inductively Coupled Plasma Optical Emission Spectroscopy (ICP-OES) was used to measure the metal content within the bacteria and corroborate the mechanism seen previously. Compound 3 and Compound 4 showed significant increases in the combination treatment relative to the compound or copper alone increasing the bacteria associated copper by 100 x over the control (Figures 3A–D). Thus, Compound 3 and Compound 4 facilitate copper entry into the bacteria.

### DsRed2 offers a novel way to determine relative affinity copper values

**Figure 4:**
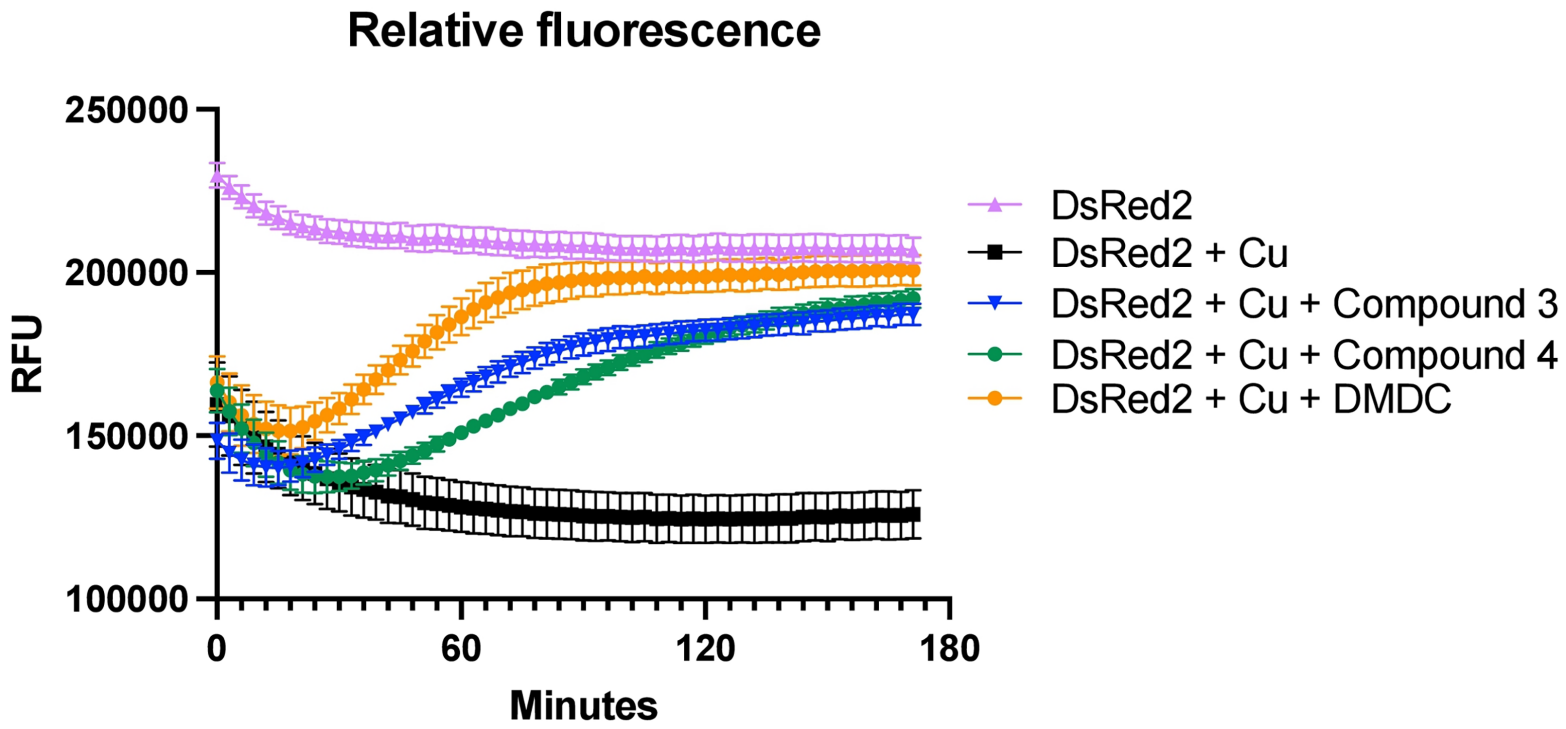
Chelation ability of compounds and DMDC for copper relative to DsRed2. Restoration of DsRed2 fluorescence treated with 100 μM copper and the different chelating compounds was measured and compared to a DsRed2-only control. Compounds were added at 400 μM. The graph shows the standard error of the mean of three independent experiments.

Determining copper affinity is notoriously difficult due to relatively high-affinity values. Thus, the ability to at least compare the binding rates of the compounds to DMDC using DsRed2, a protein that has its fluorescence quenched by copper, was pursued. The stoichiometry of DMDC to copper is 2:1 (Lei et al., 2015); this premise was used to provide the compounds in excess for the chelation assays. Using the compounds to compete for copper to reverse the quenching of copper to DsRed2, initial hierarchies in their ability to chelate copper and restore fluorescence were found (Figure 4). Interestingly, bactericidal compounds Compound 3 and Compound 4 exhibited more prolonged periods to quench DsRed2 as compared to parent compound DMDC. All had an initial dip implying that DsRed2 bound copper initially, but then the compounds took varying amount of time to equilibrate. Thus, while relative affinities and compound-mediated copper influx could play a role in the overall effectiveness of the antibiotic capacity of the compound, this process is not linear and likely highly dependent on the microbe-of-study’s phenotype and ability to flux metal in and out of the organism.

### The effect of zinc and manganese on the metal dependent toxicity of Compound 3 and 4

**Figure 5:**
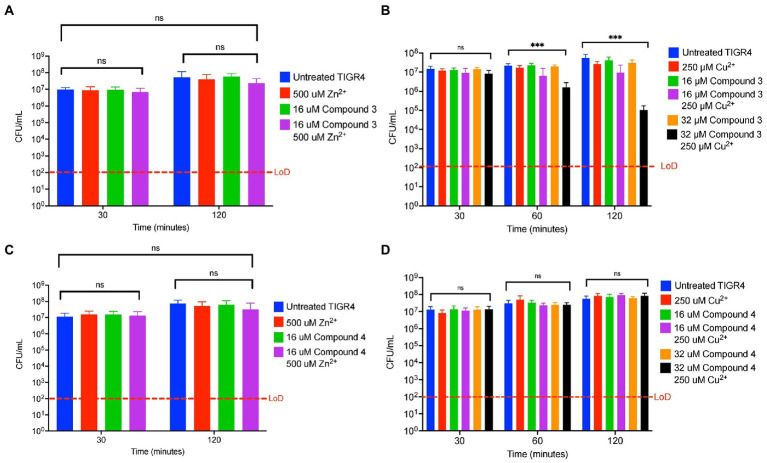
Compound 3 and Compound 4 display zinc-dependent toxicity, and the copper-dependent toxicity of Compound 3 can be rescued by Mn^2+^ supplementation. **(A)** Killing curve assay of *Streptococcus pneumoniae* TIGR4 strain starting with an inoculum of 9.9 x 10^6^ CFU/ml in M17 media supplemented with indicated concentrations of zinc sulfate and/or Compound 3. **(B)** Killing curve assay of *S. pneumoniae* TIGR4 strain starting with an inoculum of 9.0 x 10^6^ CFU/ml in M17 media supplemented with 500 μM Mn^2+^ and the indicated copper sulfate concentrations and/or Compound 3. **(C)** Killing curve assay of *S. pneumoniae* TIGR4 strain starting with an inoculum of 1.2 x 10^7^ CFU/ml in M17 media supplemented with indicated concentrations of zinc sulfate and/or Compound 4. **(D)** Killing curve assay of *S. pneumoniae* TIGR4 strain starting with an inoculum of 1.1 x 10^7^ CFU/ml in M17 media supplemented with 500 μM Mn^2+^ and the indicated concentrations of copper sulfate and/or Compound 4. Recovery of bacterial CFU/ml to that of untreated bacteria is seen at *t* = 120 minutes, with no statistically significant difference between untreated and combination treatment. All bars indicate mean ± SD with *n* = 9 across 3 independent replicates. Statistical differences were measured by one way ANOVA; ns, not significant, *p < 0.05, **p < 0.01, ***p < 0.001, and ****p < 0.0001.

In a targeted small-molecule screen that identified DMDC as a potent copper-dependent antibiotic, combinations of many divalent metals with DMDC were investigated to determine if other metals can display toxicity (Menghani et al., 2021). To test the zinc-dependent toxicity with the derivatives, killing curves were performed using TIGR4 against combinations of zinc, Compound 3 or Compound 4, and combinations of compound + zinc. It was observed that the combination of 500 μM Zn^2+^ + 16 μM Compound 3 is bacteriostatic in that there was no significant growth between the untreated growth at 30 min compared to the treated grown at 120 min (Figure 5A). Further, by virtue of still seeing copper dependent toxicity and bacterial killing, it was observed that adding 500 μM Mn^2+^ does not rescue the copper-dependent toxicity seen with 250 μM Cu^2+^ + 32 μM Compound 3 (Figure 5B). These experiments were repeated for Compound 4 (Figures 5C,D). While 500 μM Zn^2+^ + 16 μM Compound 4 was found to be bacteriostatic, strikingly, the addition of 500 μM Mn^2+^ rescued the copper-dependent toxicity seen with 250 μM Cu^2+^ + 32 μM Compound 4 (Figures 5C,D). From these data, it can be concluded that Compound 3 and Compound 4 are dithiocarbamate compounds whose copper-dependent toxicity may be mediated by facilitating mismetallation events within the bacterium but potentially at different affinities as seen in Figure 4.

### Compound 3 reduces bacterial burden in host

**Figure 6:**
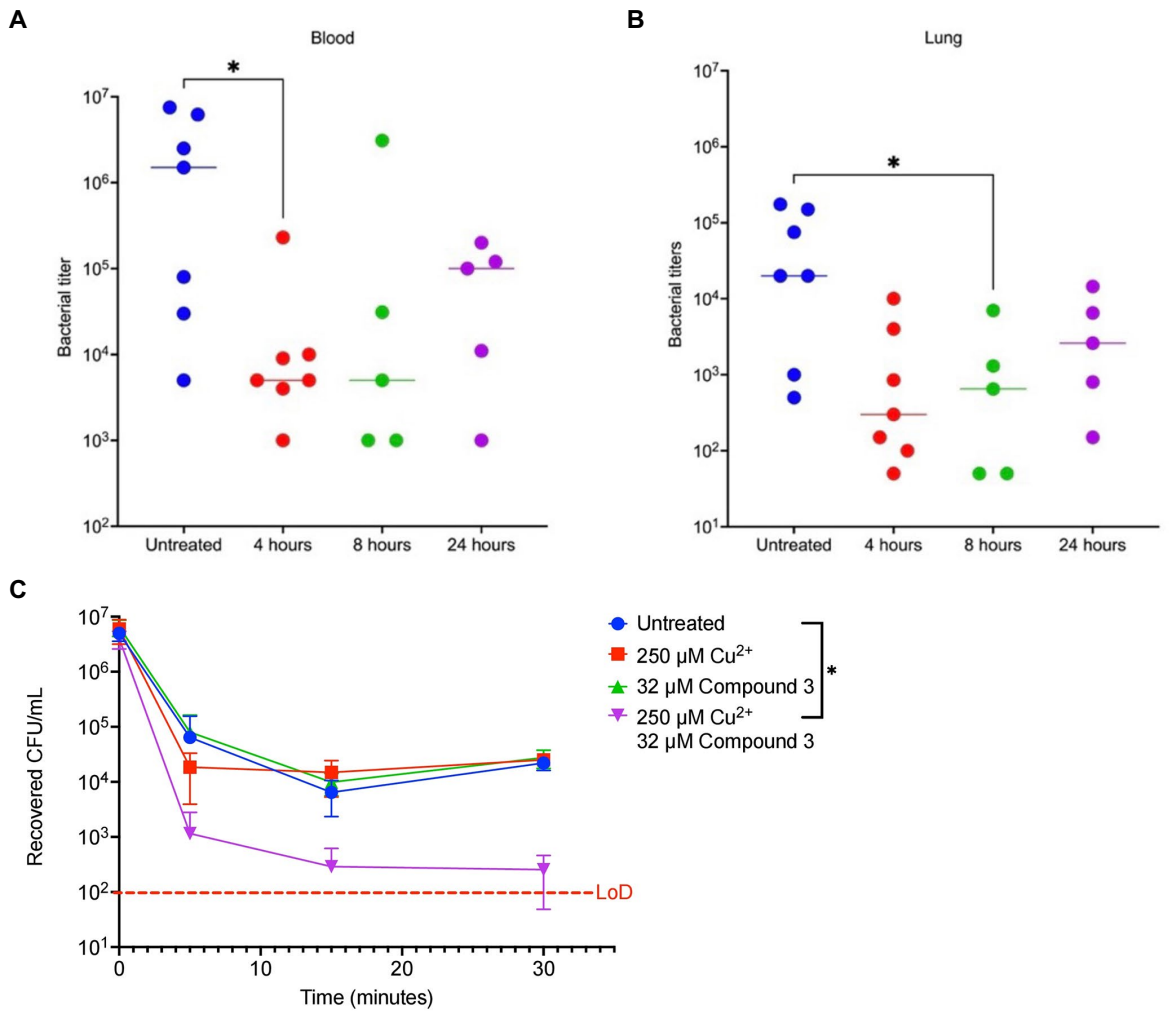
Compound 3 is an effective antibiotic *in vivo* against a murine Streptococcus pneumoniae infection model. Groups of 8-week-old female BALB/c mice (*n* = 5–7) were infected with bacteria at *t* = 0 and treated with Compound 3 at 4-, 8-, or 24-h post-infection or were untreated. At 48 h post-infection, animals were sacrificed, and blood **(A)** or lung **(B,D)** bacterial titers were measured. **(A,B)** show Compound 3 treatment reduces bacterial titers in the blood and lungs, respectively. Mann–Whitney Wilcoxon rank sum tests were used to measure statistical significance at a *p*-value of <0.05 (*) with no statistical difference noted (ns). The bar within the data set represents the median. **(C)** *Streptococcus pneumoniae* TIGR4 bacteria that were treated with indicated combinations of Cu^2+^ and Compound 3 were co-cultured with activated J774A.1 macrophages. The initial inoculum of bacteria added to the macrophages was 7.3 × 10^6^ CFU/ml (following a 15-min incubation with indicted conditions), for an MOI of 10. There is a statically significant decrease in recovered CFU/ml between the untreated bacteria and Cu^2+^ + Compound 3-treated bacteria at *t* = 5 min. At this timepoint, combination-treated bacteria were significantly cleared by the macrophages, indicating a rapid *post hoc* bactericidal killing capacity. All bars represent mean ± standard deviation (SD) with *n* = 12 across 3 independent replicates.

To directly test if the two compounds were promising antibiotics to progress along the drug discovery pipeline, they were tested in a murine pneumonia model previously used by our lab (Menghani et al., 2021). Mice were given a 100% lethal dose (LD_100_) of *S. pneumoniae* TIGR4, then at 8 h later were given Compound 3 or 4. Then, the bacterial burden was measured after 48 h. A significant decrease in bacterial titers in the blood and lungs 48 h post-infection in 8-week-old BALB/c mice given 25 μl of 10 mM Compound 3 was observed (approximately 1.6 mg/kg; Figures 6A,B). Compound 3 was also given at 4 and 24 h and observed 2 log decrease at 4 h, and reduction at 24 h, but not a significant one. No significant decrease was noted for mice at 8 h given the same dosage of Compound 4 (Supplementary Figures 1A,B). Further, as previously observed with DMDC and copper (Menghani et al., 2022), the combination treatment of Compound 3 plus copper also made the pneumococcus more susceptible to activated macrophage mediated killing *via* a macrophage killing assay as compared to no treatment, copper, or Compound 3 alone (Figure 6C). These data show that Compound 3 is a promising antibiotic against *S. pneumoniae* TIGR4 with efficacy *in vivo*.

### DMDC derivatives provide copper dependent toxicity against additional *Streptococcus pneumoniae* strains and other *streptococcal* species

**Figure 7:**
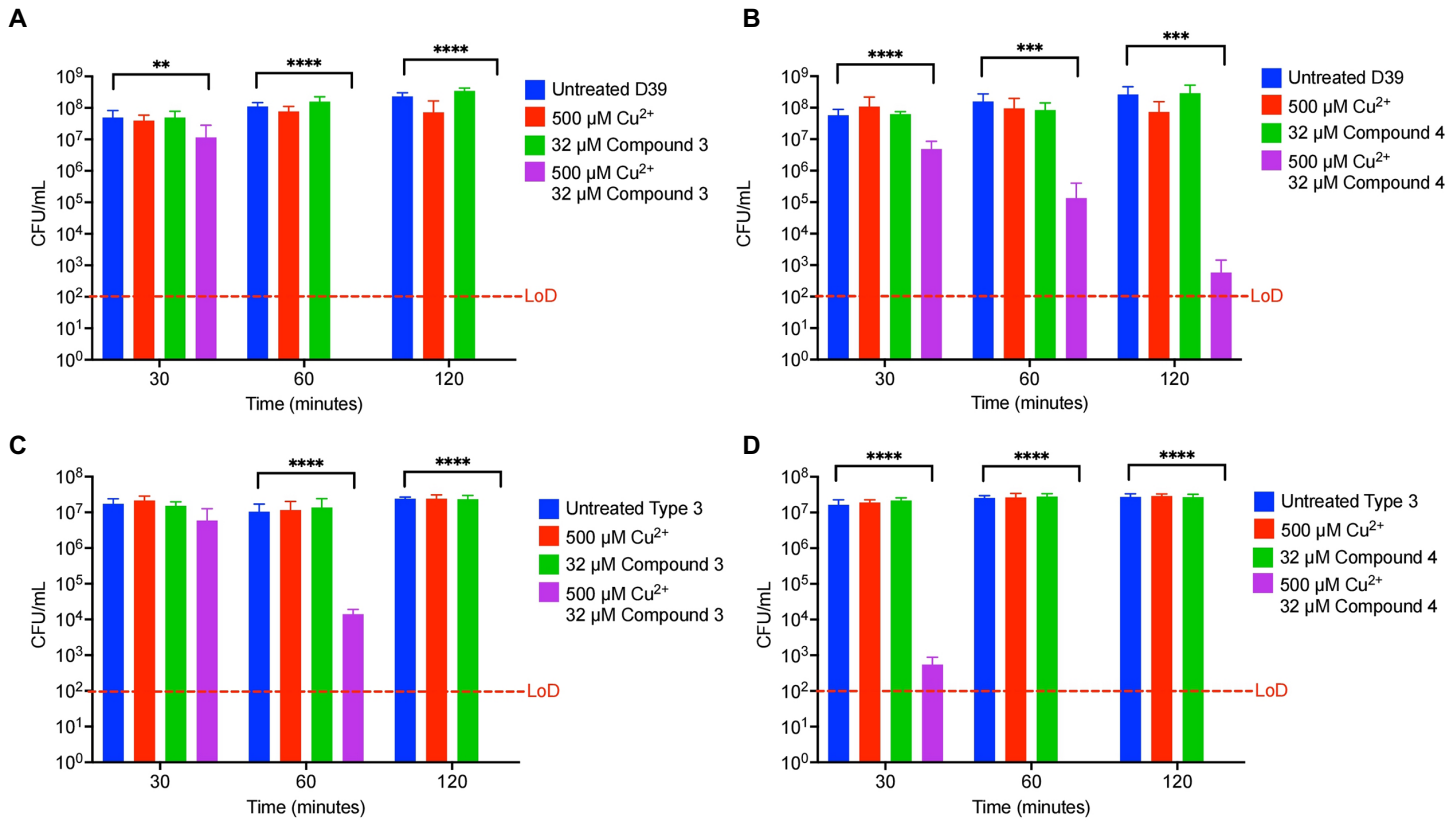
Compound 3 and Compound 4 are broadly antibiotic against multiple strains of *Streptococcus pneumoniae*. **(A)** Killing curve assay of *S. pneumoniae* D39 strain starting with an inoculum of 5.9 × 10^7^ CFU/ml in M17 media supplemented with indicated concentrations of copper sulfate and/or Compound 3. **(B)** Killing curve assay of *S. pneumoniae* D39 strain starting with an inoculum of 1.7 × 10^7^ CFU/ml in M17 media supplemented with indicated concentrations of copper sulfate and/or Compound 4. **(C)** Killing curve assay of *S. pneumoniae* 6,303 strain starting with an inoculum of 2.3 × 10^7^ CFU/ml in M17 media supplemented with indicated concentrations of copper sulfate and/or Compound 3. **(D)** Killing curve assay of *S. pneumoniae* 6,303 strain starting with an inoculum of 2.7 × 10^7^ CFU/ml in M17 media supplemented with indicated concentrations of copper sulfate and/or Compound 4. All bars indicate mean ± SD with *n* = 8–9 across 3 independent replicates. Statistical differences were measured by one way ANOVA; ns, not significant, *p < 0.05, **p < 0.01, ***p < 0.001, and ****p < 0.0001.

To determine if Compound 3 and Compound 4 are antibiotics that can be widely used against *S. pneumoniae*, killing curves were performed with these compounds against the D39 strain (serotype 2) and a serotype type 3 strain that is highly encapsulated (ATCC® 6,303™). These strains offer a small by representative sample of the more than 100 serotypes that exist for the pneumococcus. Compound 3 and Compound 4 were bactericidal in combination with copper against the D39 and the Type 3 strain, and more so (by several logs) than the effects of DMDC versus these strains as seen previously (Figures 7A–D) and to a greater degree than with TIGR4 at the 30, 60 and 120 min time point (Figure 2; Menghani et al., 2021). The intrabacterial concentration of copper with Compounds 3 and 4 against D39 and the type 3 strain was also measured. Because increased killing as compared to TIGR4 at the same concentrations (32 μM compound and 500 μM copper) was observed, lower respective concentrations (8 μM Compound and 125 μM copper) to make sure the bacteria were still viable at the time of the measurement were used. Compound 4 increased copper uptake in D39 and the type 3 strain to a similar degree as with TIGR4 (Supplementary Figures 2A–D). At these concentrations, only Compound 3 showed increased concentration of copper inside of the type 3 strain, and not D39 (Supplementary Figures 2A–D). However, not seeing the increase likely has more to do with the concentration used so viable cells were being compared.

**Figure 8:**
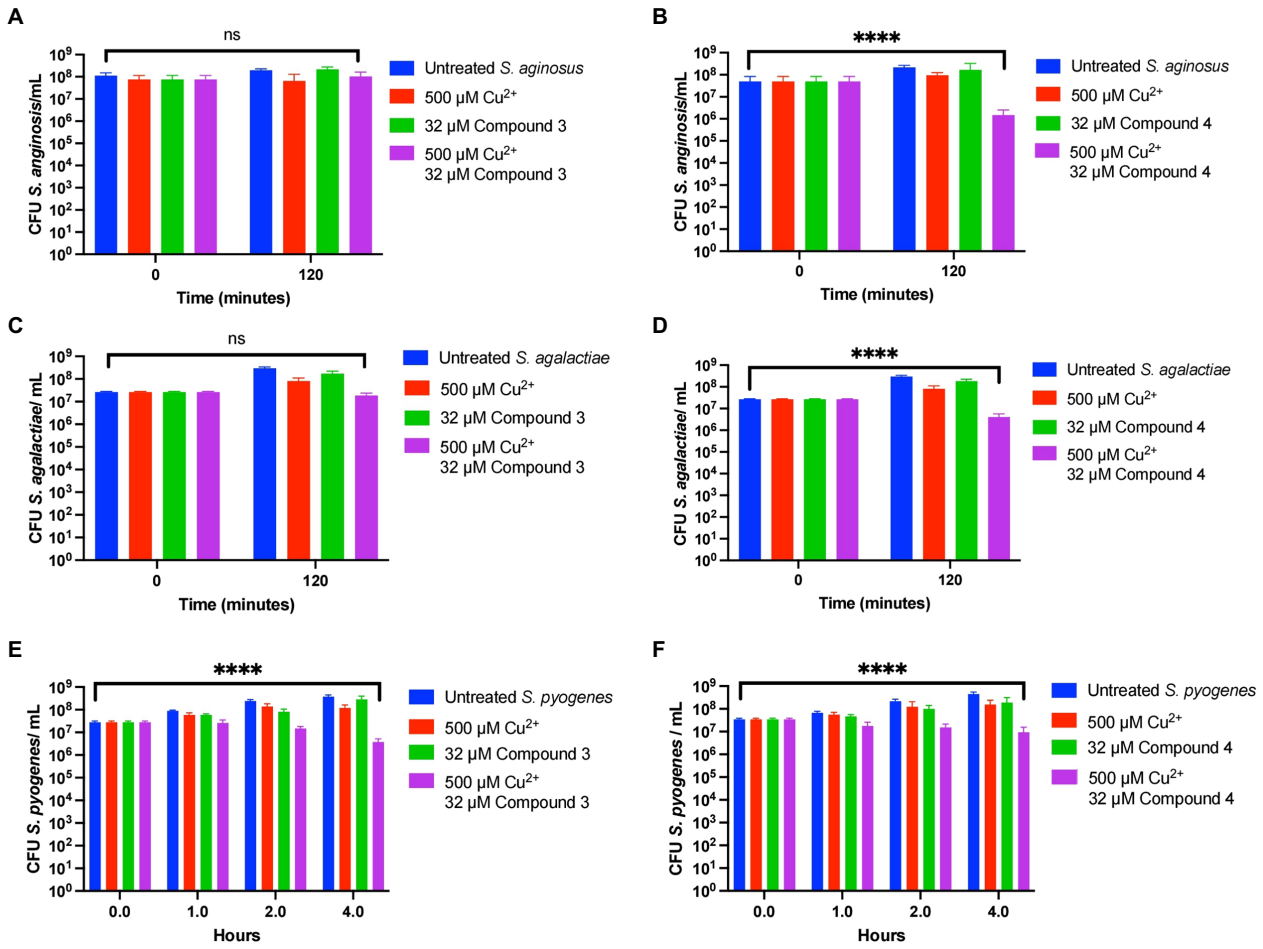
Compound 3 and Compound 4 have ranges of antibiotic activity against multiple species of *Streptococcus*. Killing curve assays depicting the effects of Compound 3 and Compound 4 against *Streptococcus anginosus* (**A,B**, respectively), *Streptococcus agalactiae* (**C,D** respectively), and *Streptococcus pyogenes* (**E,F** respectively). All bars indicate mean ± SD with *n* ≥ 3 across 3 independent replicates. Statistical differences were measured by one way ANOVA; ns, not significant, *p < 0.05, **p < 0.01, ***p < 0.001, and ****p < 0.0001.

Lastly, Compound 3 and Compound 4 were tested against *S. anginosus*, *S. pyogenes*, and *S. agalactiae*. Interestingly, there was variance between how each compound affected the survival of the *Streptococcal* species tested. Compound 3 only showed killing capacity vs. *S. pyogenes* killing roughly a log of bacteria in 4 h while the untreated grew roughly a log in this amount of time (Figures 8A, C, E). In contrast, Compound 4 demonstrated killing ability of all the tested strains killing roughly one log in each case (Figures 8B, D, F). Taken together, derivatizing DMDC is a viable strategy to targeting different strains of pneumococcus and other *Streptococcal* species, however, there is variance to the bacteria that warrants investigation to determine what makes a copper-based antimicrobial robust against one strain or species versus another.

## Discussion

The goal of this study was to test the antimicrobial efficacy of compounds derived from *N,N*-dimethyldithiocarbamate (DMDC). DMDC was previously identified as a potent copper-dependent antibiotic against *S. pneumoniae*, *S. aureus*, *C. immitis*, and *S. mansoni*, and here, was effective against other pathogenic streptococcal species (Figure 1; Menghani et al., 2021). Derivatives Compound 3 and Compound 4 showed *in vitro* inhibition in growth curves against TIGR4 while Compound 1, Compound 2, and Compound 5 had no effect (Figure 2; Table 1). Further, Compounds 3 and 4 had seemingly increased killing capacity after 30 min compared to DMDC, which did not start showing killing until the 60-min time point, thus indicating potential advancement in modifying dithiocarbamates as a copper dependent antimicrobial (Menghani et al., 2022). After identifying that these DMDC derivatives could force copper into the bacteria and chelate copper, a reduced bacterial burden during a murine respiratory infection using Compound 3 was observed (Figures 3–5). Further, the combination of Compound 3 and copper made the pneumococcus more susceptible to activated macrophage mediated killing (Figure 6C). It was also tested and confirmed that Compound 3 and Compound 4 were bactericidal against the D39 strain (which has a Type 2 capsule) and against the *S. pneumoniae* Type 3 strain (ATCC® 6,303™) (Figure 7). Compound 3 and Compound 4 displayed copper-dependent toxicity as an antibiotic against multiple strains of *S. pneumoniae* in mixed but encouraging efficacy for *S. pyogenes, S. agalactiae,* and *S. anginosus* (Figures 7, 8). It was hypothesized that there may be some initial interactions with the capsule that may be relating to the compounds speed of efficacy and overall efficacy.

It has been shown in previous studies utilizing murine models of infection that the concentration of copper increases within the blood and lungs up to four-fold (Honsa et al., 2013). Contributing to the increase in copper concentration within the lungs and blood is likely the upregulation of the acute-phase reactant ceruloplasmin, a copper-containing protein (Culbertson et al., 2020). With this increase in copper within the blood, it follows that the *in vivo* administration of dithiocarbamates such as Compound 3 and DMDC may be able to mimic the *in vitro* copper-dependent antimicrobial effect. Further investigation into the dithiocarbamates mechanism (s) of action are areas of active investigation.

Investigation of the use of compounds in the dithiocarbamate class has grown over the last few years. The anionic CS_2_ group characteristic of dithiocarbamates facilitates complex formation with transition metal cations and enzyme inhibitory activity (Hogarth, 2012). Recent advancements have shown the activity of dithiocarbamate derivatives as potent anticancer, antifungal, anti-neurodegenerative, and anti-inflammatory drugs (Shinde et al., 2020). Potent anticancer dithiocarbamates have been identified with a variety of mechanisms of action, including DNA intercalation, inhibition of DNA topoisomerase I and II, and inhibition of other essential kinases (Kamal et al., 2015). Several studies have found diethyldithiocarbamate (DEDC) to be a potent antifungal, antibiotic, and antiparasitic agent (Husain et al., 2011; Wang et al., 2018; Ghosh et al., 2020). One potential mechanism of action by which dithiocarbamate compounds can execute antibiotic activity is through transporting divalent metal ions into bacterial cells. In a study by Lanke et al., the authors show that a dithiocarbamate compound (pyrrolidine dithiocarbamate) causes an influx of Zn^2+^ ions into HeLa cells as a mechanism of action for its zinc-dependent antiviral activity against picornavirus (Lanke et al., 2007). It was hypothesized that DMDC, Compound 3, and Compound 4 exhibit similar functions, aiding transport of divalent copper ions into the bacterial cell, but not so much with zinc (Figures 5A, C). As an influx in intra-bacterial copper ions builds up due to dithiocarbamates shuttling them into the bacterium, mismetallation can cause bacterial enzymes to function at lower rates and lead to bacterial death. Accordingly, given that manganese was only able to rescue Compound 4 and not Compound 3 may be more related to the overall affinity of these compounds for copper (Figures 4, 5B, D).

Given the differences in how the derivatives function differently against the different species, a reasonable follow up to this study is to leverage (1) make additional derivatives, (2) use these derivatives against other pathogenic bacteria, and (3) determine the rules that govern the efficacy of a given ionophore. Point 3 can likely be accomplished by understanding the metal flux of copper into different organisms as a function of the ionophores’ capacity to chelate copper and the homology of metal importers that could be functioning to take in the complex.

Overall, data here supports that from a small-molecule screen of compounds derived from DMDC, that Compound 3 and Compound 4 are potent copper-dependent antibiotics against several strains of *S. pneumoniae* and pathogenic Streptococcal species. In addition, it was shown that both of those compounds exhibit zinc-dependent toxicity, and one of the compounds, Compound 3, exhibits potent *in vivo* efficacy as an antibiotic for aiding murine *S. pneumoniae* clearance. Further investigation into dithiocarbamate derivatives warrants further study for developing novel antimicrobials in the fight against antimicrobial-resistant pathogens.

## Data availability statement

The raw data supporting the conclusions of this article will be made available by the authors, without undue reservation.

## Ethics statement

The animal study was reviewed and approved by IACUC at the University of Arizona.

## Author contributions

MJ, SM, and YS-R contributed to the conception and design of the study. SM, YS-R, CP, HO’B, KO, MD, NH, and RH provided experimental design. RL, FG, and WW provided compound design and quality control. MJ and SM wrote the manuscript. All authors contributed to the article and approved the submitted version.

## Funding

This study was supported by NIH grant 1R35128653. Additionally, SM’s training was supported by an F30 Ruth L. Kirschstein Individual Predoctoral NRSA Fellowship from NIGMS (5F30GM139246-02). DsRed2-pBAD was a gift to MJ (Addgene plasmid # 54608; http://n2t.net/addgene:54608; RRID:Addgene_54608).

## Conflict of interest

The authors declare that the research was conducted in the absence of any commercial or financial relationships that could be construed as a potential conflict of interest.

## Publisher’s note

All claims expressed in this article are solely those of the authors and do not necessarily represent those of their affiliated organizations, or those of the publisher, the editors and the reviewers. Any product that may be evaluated in this article, or claim that may be made by its manufacturer, is not guaranteed or endorsed by the publisher.
