# Supplementary material for: Novel dithiocarbamate derivatives are effective copper-dependent antimicrobials against Streptococcal species
Source: Front Microbiol. 2023 Jan 20;13:1099330. doi: 10.3389/fmicb.2022.1099330 (PMC9894897; doi:10.3389/fmicb.2022.1099330)
Supplement: Supplementary file 1 [file Data_Sheet_1.docx]

## Supplemental Data for Novel dithiocarbamate derivatives are effective copper-dependent antimicrobials against *Streptococcal* species

Sanjay V. Menghani^1,2^, Yamil Sanchez-Rosario^1^, Chansorena Pok^1,3^, Renshuai Liu^4^, Feng Gao^4^, Henrik O’Brien^1^, Miranda J. Neubert^1^, Klariza Ochoa^1^, Meredythe Durckel^1^, Riley D. Hellinger^1,2^, Nadia Hackett^1^, Wei Wang^4^, Michael D. L. Johnson^1,5,6,7^

^1^Department of Immunobiology

University of Arizona College of Medicine - Tucson

Tucson, AZ, 85724

^2^Medical Scientist Training MD-PhD Program (MSTP)

University of Arizona College of Medicine - Tucson

Tucson, AZ, 85724

^3^Current address

Department of Microbial Pathogens and Immunity

Rush University

Chicago, IL, 60612

^4^Department of Pharmacology and Toxicology

R. Ken Coit College of Pharmacy, University of Arizona

Tucson, AZ 85721

^5^Valley Fever Center for Excellence

University of Arizona College of Medicine - Tucson

Tucson, AZ, 85724

^6^BIO5 Institute

University of Arizona College of Medicine - Tucson

Tucson, AZ, 85724

^7^Asthma and Airway Disease Research Center

University of Arizona College of Medicine - Tucson

Tucson, AZ, 85724

Corresponding Author: Michael D. L. Johnson

University of Arizona

1656 E. Mabel St. / P.O. Box 245221 / MRB 213 (office)

Tucson, AZ 85724

Tel: 520-626-3779 / Fax: 520-626-2100

mdljohnson@arizona.edu

Supplemental Table 1 - List of bacterial strains used in this study

**Supplemental Figure 1 -** **Compound 4 is not an effective antibiotic in vivo** **against the TIGR4 strain of *S. pneumonaie***

Groups of 8-week-old female BALB/c mice were infected with bacteria at t = 0 and treated with Compound 4 at 8-hours post-infection or were untreated. At 48 hours post-infection, animals were sacrificed, and blood (A) or lung (B) bacterial titers were measured. Mann-Whitney Wilcoxon rank sum tests were used to measure statistical significance at a P value of <0.05 (*) with no statistical difference noted (ns). The bar within the data set represents the median.

**Supplemental Figure 2 -** **Intracellular copper concentration of D39 and Type 3 after treatment with compounds 3 and 4 with copper**

Exponentially growing *S. pneumoniae* D39 (A and B) or *S. pneumoniae* TIGR4 Type 3 (C and D) bacteria were treated with the indicated compound and/or copper or left untreated for 30 minutes. ICP-OES was used to measure intracellular copper. (A) and (C) represent the moles of copper per CFU while (B) and (D) represent the fold change over the respective strains with nothing added. Experiments were performed in triplicate with statistical significance determined by an Ordinary one-way ANOVA; **p < 0.01. Each point represents 6 individual sample replicates.

Supplemental Table 1

Supplemental Figure 1

Supplemental Figure 2
